# Law and psychology must think critically about effect sizes

**DOI:** 10.1007/s44202-022-00062-2

**Published:** 2023-01-12

**Authors:** Jason M. Chin

**Affiliations:** College of Law, Australian National University, Canberra, Australia

**Keywords:** Law and psychology, Forensic psychology, Credibility revolution, Effect sizes, Public science, Implicit bias

## Abstract

This comment examines a threat to the development of law and psychology as a “public science” (i.e., one that goes beyond theory to address important issues in society), a failure to think critically about effect sizes. Effect sizes estimate the strength or magnitude of the relationship between variables and therefore can help decision makers understand whether scientific results are relevant to some legal or policy outcome. Accordingly, I suggest that those conducting and reporting law and psychology research should: (1) justify why observed effect sizes are meaningful and report them candidly and transparently, (2) scrutinize effect sizes to determine if they are plausible, and (3) plan studies such that they fit with the researchers’ inferential goals. I explore these points by way of case studies on influential law and psychology studies, such as implicit bias in the courtroom. I end with suggestions for implementing my recommendations, including a metaresearch agenda for law and psychology.

Discussions about psychology’s role as a science that aims to address “pressing issues in society” (i.e., public psychology, [1]; see also [2]) have taken on newfound poignance in recent years. Beyond the importance of issues such as the government’s behavioral science response to the COVID-19 pandemic [3], many conversations revolve around psychology’s ongoing “credibility revolution” [2, 4]. That revolution involves the field studying its own processes (i.e., metaresearch) and adopting more rigorous and transparent methods, such as prospective registration (i.e., preregistration) [5] and open data and code [6, 7]. These developments are especially pressing in law and psychology,^1^ a subfield of psychology that regularly informs criminal justice outcomes and public policy [9]. For instance, law and psychology research is used to inform guidelines for taking witness statements and eyewitness identifications. Yet, sustained conversations about developing a public law and psychology appear to be rare [10–14] and have largely not focused on effect sizes (but see, [13, 14]).

In this comment, I will examine a key methodological threat to the development of a public law and psychology—a failure to think critically about effect sizes. That is, it appears to not yet be common practice in law and psychology for researchers to (1) provide empirical or at least verifiable support for why effect sizes might matter, (2) to scrutinize effect sizes that are incredibly large, and (3) to plan studies [15] such that they are informative of whether a meaningful effect exists (i.e., practical relevance, see [13, 14]). I analyze these issues with respect to influential studies in law and psychology, and then provide discipline specific guidance for moving forward.

## Justify the meaningfulness of effect sizes

As an applied field, law and psychology’s remit goes beyond exploring the theoretical relations between psychological constructs and behavior, to building knowledge about whether some observed relationship matters (see [13, 14]). For example, some intervention aimed at improving a legal outcome might be consistently observable and of theoretical interest. However, it may possess an effect size so small that it is unlikely to be worth the time and cost in implementing it.

Consider, for example, the widely discussed issue of implicit bias (i.e., automatic associations between some social group and an evaluation, such as associating a group with violence or aggression) affecting legal outcomes [16, 17]. In a recent article written for judges, Kang [18] summarized meta-analyses finding a generally small relationship (“The range of r values goes from 0.24 down to 0.10”, [18]) between implicit bias measures and discriminatory behavior. He did not, however, acknowledge the overstatement of effect sizes in small, non-preregistered studies (such studies provide effect sizes twice as large as compared to large, preregistered replications, see [19, 20]) that would make the estimates he provided even smaller with less obvious implications for the legal system.

Kang also laid out an unqualified case for effect size accumulation whereby a lawyer subject to implicit bias would be less likely to make it to partner over the course of their career, even if there was just 1% increased chance of attrition due to implicit bias at any given time point: “After eight years (or 96 cuts), it turns out that Greg's partnership chance is 38.1% (0.99^96 = 0.381). Brandie’s is only 14.4% (0.98^96 = 0.144)” [18]. In other words, he posits that these small psychological effects build across time and people [21, 22] such that even if it they are small on an individual level, their effects can be larger on a macro level.

This legal-scientific communication about effect sizes—written for a judicial audience—could be improved in many ways. As Lewis and Wai (2, p. 1246) note, scientific communication requires humility and transparency: “scientific communication should […] make clear to your audience both what you know (and do not know), and how you know it.” To follow Lewis and Wai’s prescription, psychologists communicating with legal actors about topics such as implicit bias should acknowledge evidence that reported effect sizes tend to be inflated. In Lewis and Wai’s terms, this makes clear what is still unknown (precise effect size estimates) and why (bias in research and reporting). Similarly, statements that effect sizes accumulate should acknowledge that there is no empirical research supporting this in many contexts [23] and provide verifiable lines of reasoning explaining why they may or may not accumulate [24]. Communication in this style gives life to more general calls for legal psychologists to take caution when communicating with nonscientists [11].^2^

## Scrutinize incredibly large effect sizes

Just as small effect sizes raise questions that should be addressed, so do incredibly large effects. A law and psychology example of an incredibly large effect can be found in an influential study of parole decisions made by Israeli judges [25, 26], cited 1,653 times as of this writing.^3^ This study found that favorable parole decisions dropped from about 65% earlier in the day to nearly 0% just before lunch and then returned to 65% after lunch (and other meal breaks). This is a very large effect (Cohen’s *d* = 2). In subsequent commentaries, Lakens [27, 28] contextualized this effect against others in psychology and against its real-world plausibility, concluding it was incredible: “There are hardly any effects in psychology this large, let alone effects of mood or rest on decision making. If mental depletion actually has such a huge real-life impact, society would basically fall into complete chaos just before lunch break every day” [28].

Lakens’ critique has found support in other analyses [29–31]. For instance, simulations find that scheduling shorter matters before lunch breaks accounts for the hungriness effect [30]. Similarly, through interviewing court staff from the original study, Weinshall-Margel and Shapard [31] found several variables unaccounted for in the original analyses, such as unrepresented parties being more likely to be heard in sessions before breaks. The original authors [25] reanalyzed their data and found that the factors uncovered by Weinshall-Margel and Shapard [31] did not fully explain the “hungriness” effect, but they did not report whether controlling those variables reduced the size of the effect.

For our purposes, the hungry judges study and its fallout underscore the importance of critically appraising effect sizes and transparently discussing and reporting them. Indeed, as we saw from Lakens’ work, simply considering whether the hungry judge effect could plausibly be larger than cognate effects studied in the lab raised important questions casting doubt on the study’s conclusions. Still, many publications continue to cite the original hungry judge study uncritically (see e.g., [32–34]). As a result, it is important that authors finding effects that seem too big to be true highlight this in discussion sections so that readers can assign the proper weight to them. This is especially important in applied research, a context in which readers are often legal actors who may accept psychological findings at face value. And, at a minimum, researchers should report effect sizes and make their data available when possible. As we saw with the hungry judges study, the authors did not report the degree to which the effect sizes changed when more controls were introduced [29]—this is essential information for legal decision makers.

## Justify sample sizes to perform publicly meaningful studies

Finally, thinking critically about effect sizes is important to ensuring that law and psychology studies can inform the important public policy questions they seek to address. One example of the unfortunate consequences that flow from a failure to plan a study to find a meaningful effect size can be found in a study commissioned by Australia’s Royal Commission into Institutional Responses to Child Sexual Abuse (“RCIRCSA”) [35, 36]. That study sought to determine whether there was a biasing effect of “joining” trials such that juries hear and decide about allegations of sexual assault against multiple complainants purported to be committed by the accused [36]. If joining trials does not increase bias, then that is a good reason to reform the rules that allow for joined trials. And indeed, the authors found no statistically significant effect [36]. This null effect has informed a changed in evidence law in one large Australian state [37].

Closer attention to effect sizes would have made the RCIRCSA more useful. Specifically, the mere absence of a statistically significant effect tells us little about whether a publicly meaningful effect exists. Indeed, the authors reported that they powered the study to find an effect size “determined based on the magnitude of effects observed in past studies” [36] and did not report those calculations. Beyond this lack of transparency, there are two problems with powering the RCIRCA study to find previously reported effects. First, as we saw above, effect sizes in the literature are often overstated and so it is likely the RCIRCSA study was underpowered to find the effect they thought they were seeking. But, more importantly, the sample size justification did not fit with the RCIRCSA’s inferential goal. Powering a study to find an effect size estimated from previous research is a justification based on the “expected effect size” [38]. In other words, the researchers are powering a study to find an effect of the size that research and theory predicts. However, that justification is only weakly (or perhaps not at all) related to the goal of determining whether it is safe to join trials.

The preferable strategy would have been for the experimenters to meet with the RCIRCSA’s legal stakeholders to determine the smallest effect size that they consider to be practically important. For example, are courts and criminal justice practitioners willing to tolerate 5 or 10% more convictions in joint trials (all else equal) to improve the efficiency of justice system and other competing policy demands? Determining this—the smallest effect size of interest—is a challenge. In one area of legal psychology, false memory research, respondents to a survey could not agree on the smallest effect size of interest and many conflated it with statistical significance [15]. But the task is easier and worth undertaking in publicly commissioned research with a clear goal of informing a specific change in law (versus studying false memories generally).

## Towards a public law and psychology

These challenges with effect sizes in law and psychology help illuminate a path forward for both those who communicate with legal stakeholders and those who conduct research. Starting with communication, individuals and institutions addressing legal actors must not assume that those actors will be aware of the uncertainties surrounding effect size estimates (see e.g., [11]). That is, we cannot assume that judges, policymakers, and the like possess the scientific knowhow to do anything more than take a reported effect size at face value. Effect size inflation [19, 20], and the numerous reasons that effect sizes may not accumulate in the wild [24] are not obviously common knowledge. While it is undeniably important to inform legal actors that an effect *may* be occurring, to *assign a size* to it without alerting them about the uncertainty surrounding that size is dangerous.

Researchers—when conducting research—also have an important role to play. Perhaps most fundamentally, law and psychology researchers must transparently report their effect sizes, their sample size justifications (which often hinge on an effect size), and any calculations underlying that work [38]. As we saw [29], such reporting does not always occur, yet is important in evaluating the legal and policy implications of psychological research. Similarly, researchers should candidly report both how effect sizes may or may not accumulate in the across time and people. By way of analogy, psychological researchers have prescribed [39] constraints on generality statements for research, which may be especially useful for lay readers to understand why a study may not apply to a context or population. The same level of candor and transparency should also be routine when presenting effect sizes in applied research.

Finally, law and psychology researchers should consider engaging in increased metaresearch—research on its own methods and processes [40]. This work is underway, including the previously discussed survey of false memory researchers about the smallest effect size of interest in their work [15] and scrutiny of the hungry judges study finding several reasons its findings were likely overstated (e.g., [29]). However, much more is needed and can generally follow the discussion above. For instance, large scale preregistered replication projects of foundational studies can help provide more precise estimates of effect sizes and can be used as a basis to begin to estimate heterogeneity of effects across populations and contexts [12, 41]. This work can help replace more impressionistic metaresearch in law and psychology which often relies on subjective judgments of researchers about what work has reached “general acceptance” (see e.g., [42]). It can also supplement this comment, which has relied on case studies rather than a systematic survey of the literature. And, to capitalize on these efforts [40], researchers should conduct regular audits (in psychology generally, see e.g., [43]) of reporting practices in law and psychology to see if reform efforts are working—that is, if researchers and practitioners actually begin to report effect sizes more transparently and more cautiously.

## Data Availability

There are no data or code related to this Comment.

## Footnotes

1 This article will collapse several fields in which psychology informs law and policy into the term “law and psychology”. See Neal [8] for a more careful dissection of the various subfields encompassed by law and psychology.

2 A relevant quote from Ellsworth [11] is: “We have to recognize that research on social issues gets communicated to a much broader audience than basic research. We can assume that our colleagues will be skeptical about our claims in the discussion section and they’ll evaluate them in terms of what they read in the methods sections and the results that we actually got. People that are not scientists—legislators, judges, reporters, the public—are more likely to read only the introduction and discussion”.

3 https://scholar.google.com/scholar?cites=2591465710359909216&as_sdt=2005&sciodt=0,5&hl=en.
